# Effect of the metal ion-induced carbonylation modification of mitochondrial membrane channel protein VDAC on cell vitality, seedling growth and seed aging

**DOI:** 10.3389/fpls.2023.1138781

**Published:** 2023-05-31

**Authors:** Ying Li, Chang Liu, Manyao Qi, Tiantian Ye, Ying Kang, Yu Wang, Xiaofeng Wang, Hua Xue

**Affiliations:** State Key Laboratory of Tree Genetics and Breeding, National Engineering Research Center of Tree Breeding and Ecological Remediation, College of Biological Sciences and Biotechnology, Beijing Forestry University, Beijing, China

**Keywords:** metal-binding protein, metal-catalyzed oxidation (MCO), protein carbonylation, voltage-dependent anion channel (VDAC), *Ulmus pumila* L., seed aging

## Abstract

**Introduction:**

Seeds are the most important carrier of germplasm preservation. However, an irreversible decrease in vigor can occur after the maturation of seeds, denoted as seed aging. Mitochondrion is a crucial organelle in initiation programmed cell death during seed aging. However, the underlying mechanism remains unclear.

**Methods:**

Our previous proteome study found that 13 mitochondria proteins underwent carbonylation modification during the aging of *Ulmus pumila* L. (Up) seeds. This study detected metal binding proteins through immobilized metal affinity chromatography (IMAC), indicating that metal binding proteins in mitochondria are the main targets of carbonization during seed aging. Biochemistry, molecular and cellular biology methods were adopted to detect metal-protein binding, protein modification and subcellular localization. Yeast and Arabidopsis were used to investigate the biological functions *in vivo*.

**Results and discussion:**

In IMAC assay, 12 proteins were identified as Fe^2^+/Cu^2^+/Zn^2^+ binding proteins, including mitochondrial voltage dependent anion channels (VDAC). UpVDAC showed binding abilities to all the three metal ions. His204Ala (H204A) and H219A mutated UpVDAC proteins lost their metal binding ability, and became insensitive to metal-catalyzed oxidation (MCO) induced carbonylation. The overexpression of wild-type UpVDAC made yeast cells more sensitive to oxidative stress, retarded the growth of Arabidopsis seedlings and accelerated the seed aging, while overexpression of mutated UpVDAC weakened these effects of VDAC. These results reveal the relationship between the metal binding ability and carbonylation modification, as well as the probable function of VDAC in regulating cell vitality, seedling growth and seed aging.

## 1 Introduction

Seed quality plays an important role in agricultural and forestry production, the effective conservation of genetic resources, and biodiversity protection. Seeds will experience an irreversible decrease in vitality from maturity, denoted as seed deterioration or aging. Seed aging is a common physiological phenomenon during seed storage, associated with mitochondrial alterations, programmed cell death, DNA repair, the antioxidant system, telomere length, and epigenetic regulation (Ahmed et al., 2017). Seed longevity is a complex problem determined by seed quality and storage conditions. Studies have shown that seeds of high initial vigor at low temperature and humidity can be stored for a long time, and the germination percentage decreases slowly (Solberg et al., 2020). Seed aging reduces seed vigor and affects seedling growth, plant yield, and quality at later stages. Therefore, exploring the mechanism of seed aging is of great significance.

Reactive oxygen species (ROS) are thought to play a dual role in plant biology. They are necessary for many important signaling responses, but are also toxic byproducts of aerobic metabolism. ROS is considered to be the result of physiological or procedural pathways that trigger cell death. It may act as a signal molecule in the early stage of seed aging and trigger the process of apoptosis (Bailly et al., 2008; Mittler, 2017). Research on the morphological and dynamic changes of mitochondria proved that mitochondria are the primary source of endogenous ROS during seed aging. Following the artificial aging of soybean seeds, the activities of antioxidant enzymes and the ascorbic acid glutathione (ASC-GSH) cycle in the mitochondria were significantly reduced (Wang et al., 2015). Moreover, the ROS was observed to significantly increase, which may explain the dysfunction of mitochondria during seed aging (Xin et al., 2014). These findings suggest that the formation of mitochondrial ROS plays an important role in reducing seed vigor during aging.

Mitochondria are the center of cell energy and organic metabolisms. They are also key subcellular components to maintain metal homeostasis in cells. Many proteins in the metabolic process of mitochondria require metal ions as a cofactor to ensure normal catalytic functions, such as Mn-SOD, cytochrome, and most enzymes in the tricarboxylic acid cycle (Salvato et al., 2014). However, metal ions can also be toxic to cells. Much literature reports the ability of metals to reduce the function of the intracellular antioxidant system, resulting in the accumulation of ROS (Nouet et al., 2011; Qu et al., 2014; Valko et al., 2016). Redox-active metal ions (such as copper) catalyze the production of ROS when combined with β amyloid peptides (Cheignon et al., 2017). Iron-activated ROS has been observed to induce pyroptosis in melanoma cells *via* a Tom20-Bax-caspase-GSDME pathway (Zhou et al., 2018), while non-redox active metal Zn can inhibit mitochondrial function (Vygodina et al., 2008; Liu et al., 2014; Glińska et al., 2016). These studies have verified the toxic effects of exogenous metal ions on cells.

VDAC is a mitochondrial porin located in the outer membrane of mitochondria. It belongs to one of the two subfamilies of porin family 3, serving as the channel for metabolites to enter and exit the mitochondrion. VDAC regulates the mitochondrial function and cell survival by controlling the exchange of material and energy between the mitochondria and other organelles (Kanwar et al., 2020). VDAC is also an important element in mitochondrial apoptosis. Therefore, VDAC can also serve as a hub of signal transduction in the cell death process through interactions with other proteins or ligands (Shoshan-Barmatz et al., 2010). Studies have shown that VDAC-tubulin interaction regulates the VDAC opening, which comprehensively controls the mitochondrial metabolism, ROS production, and intracellular energy flow (Fang and Maldonado, 2018). Post-translational modification of VDAC on cysteine determined by mass spectrometry analysis is able to counteract the excess of mitochondrial ROS load (Reina et al., 2020). The amino acid sequence of VDAC is highly conserved from yeast to humans (Colombini, 2004). The three-dimensional structure of human VDAC1 protein has been investigated by new methods that combine nuclear magnetic resonance and X-ray crystallography. It shows that VDAC consists of 19 β-sheets and an N-terminal α-helix, which forms a barrel-like structure (Bayrhuber et al., 2008; Hiller et al., 2008). The barrel wall and α-helix contain multiple charged amino acid residues, and the high electric charge density provides the channel inside with the potential ability of anion transport (Ujwal et al., 2008).

Protein carbonylation is a non-enzymatic and irreversible post-translational modification induced by ROS (Tola et al., 2021). Protein carbonylation may play a role in seed dormancy release and germination. Previous research has demonstrated that many proteins are carbonylated during Arabidopsis seed germination and storage (Leymarie et al., 2012). Selective protein oxidative modification has been found in many studies on plant stress response. Some proteins in rice mitochondrial matrix treated with Cu^2+^ and H_2_O_2_ undergo selective carbonylation (Kristensen et al., 2004). In addition, fruit aging and deterioration are related to the selective carbonylation of mitochondrial proteins, and mitochondrial porin VDAC is particularly sensitive to oxidative damage (Qin et al., 2009).

Metal-catalyzed oxidation (MCO) is the most widely accepted protein oxidative damage mechanisms. Protein carbonylation was identified as the main result of MCO. Carbonylation can be formed directly by attacking the amino acid side chains of arginine (Arg), lysine (Lys), proline (Pro), histidine (His)and threonine (Thr), or by the reaction of lysine and cysteine with active carbonyl groups on carbohydrates (sugar oxidation products), lipids, or end products of lipid oxidation. This protein oxidative modification is site-specific (Maisonneuve et al., 2009; Ciacka et al., 2020). Studies have pointed out that metal-binding proteins may be a significant target for carbonylation as they are more sensitive to MCO (Stadtman, 1990). A protein is more likely to become a target of oxidative damage, particularly when the oxidation-sensitive amino acid sites are on the surface of the protein and bind with metal ions.

Previous literatures have widely applied immobilized metal affinity chromatography (IMAC) to verify the relationship between carbonylation and metal stress. Scholars used IMAC to screen proteins that interact with Cu in Arabidopsis roots, revealing that His, Met, and Cys are the primary sites for Cu binding in the conserved motifs of metalloproteins (Kung et al., 2006). Tandem mass spectrometry (MS) analysis has shown that the amino acid site near Cu binding site is prone to oxidative modification (Bridgewater et al., 2006). Researchers also employed IMAC to screen proteins in Arabidopsis that interact with Cu, Zn, and cobalt (Co) and found that the activity of these metal-binding proteins can be inhibited by related metal ions (Tan et al., 2010).

Previous study has shown that the carbonylation of VDAC during seed aging may alter its channel function (Li et al., 2017). However, its details and physiological consequence remain to be explored. In the current study, various metal-binding proteins, including mitochondrial outer membrane protein VDAC, were observed to be carbonylated during the aging process of elm seeds. Biochemical, cytological, and transgenic methods were subsequently adopted to investigate the relationship between the metal binding ability of VDAC and protein carbonylation and its role in cell vitality, seedling growth and seed aging.

## 2 Material and methods

### 2.1 Materials

Elm seeds were collected from the campus of Beijing Forestry University, with an initial germination rate of 98% and an initial water content of 0.077 g water/g dry weight. The seeds were soaked with 5% sodium hypochlorite for 10 min, washed with distilled water (ddH_2_O) three times to remove the residual disinfectant, and dried back to the initial water content at 25°C. The wild-type Arabidopsis Colombia (Col) ecotype was purchased from Arabidopsis Biological Resource Center. Arabidopsis were cultured at 25°C, 65% relative humidity with a 16-hour light/8-hour dark cycle.

The T vector pMD19-T, prokaryotic expression vector pGEX-4T-1, plant binary expression vector pBI121, and yeast expression vector pYES2/CT were stored in the laboratory.

### 2.2 Controlled deterioration treatment

The elm seeds were pretreated with Fe^2+^ (20 mM), Cu^2+^ (10 mM), Zn^2+^ (30 mM), EDTA (1 μM) or metal salts with H_2_O_2_ (10 mM) at 25°C for 4 hours, and re-dried to their original moisture content.

The CDT was performed as described previously (Hu et al., 2012) with some modification. The elm or Arabidopsis seeds were aged in a sealed jar with ddH_2_O (100% relative humidity) at 37°C. The aging process was initiated from one day of post-equilibration (PE), in which uncovered petri dishes with seeds were put above the water layer of the ageing jar. Then the seeds were transferred into sealed aluminum-foil bags (elm seeds) or eppendorf tubes (Arabidopsis seeds) to age for two or three days.

When the CDT was finished, seeds were re-dried to their original moisture content and subjected to mitochondrion separation (elm seeds) or germination tests (Arabidopsis seeds).

### 2.3 Mitochondrion separation

A total of 1,000 elm seeds were imbibed in ddH_2_O for 12 hours (50 seeds per dish, 20 dishes in total). The testa was peeled, and the seeds were washed repeatedly with distilled water until the water was clear. The seeds were then incubated with 0.1 M PBS pH 7.4, 0.01 M EDTA at 4°C for 20 min to chelate Ca^2+^ and Mg^2+^ and then with 5 volumes of mitochondrial extraction buffer (0.33 M sucrose, 0.05 M Tris-HCl pH 7.6, 0.05 M KCl, 0.01 M EDTA, 2 g/L BSA) for 30 min. Following this, the seeds were ground with extraction buffer and filtered with 8 layers of gauze. The supernatant was then retained after differential centrifugation at 3,500 g and 4°C for 10 min. The supernatant was subsequently centrifuged at 12,000 g and 4°C for 15 min and then discarded to obtain the product. A total of 5 mL extraction buffer was added to each tube to resuspend the pellet, followed by another differential centrifugation at 12,000 g and 4°C for 15 min. This was processed by the addition of 1 mL suspension buffer (0.34 M sucrose, 0.01 M Tris-HCl, 0.005 M EDTA) to resuspend the sediment gently with a brush and a pipette. In a 10 mL centrifuge tube, 40% and 23% Percoll diluted in MTE solution (mannitol 77.68 g/L, 0.005 M Tris-HCl, pH 7.6, 0.02 M EDTA) was added with a volume ratio of 1:2 from the bottom to the top to form two liquid layers, and the rude extracts of mitochondria were gently added to the top layer. After centrifugation at 40,000 g and 4°C for 1 hour, the mitochondria extracts in the lower layer (between 23% and 40% Percoll) were aspirated. This was followed by the resuspension with 10 mL of suspension buffer. The sample was subsequently centrifuged at 20,000 g and 4°C for 15 min. Finally, the sediment was resuspended in 200 μL suspension buffer and directly used or stored at -80°C after adding 5 μL DMSO.

### 2.4 Mitochondrial protein extraction

The mitochondrial sample was frozen in liquid nitrogen and thawed repeatedly for 10 cycles. Following this, 750 μL of mitochondrial extraction buffer was added and shaken on ice for 10 min. An equal volume of Tris-saturated phenol (pH 7.9) was then added and shaken at 25°C for 10 min. After centrifugation at 5,500 g and 4°C for 10 min, the upper phenol phase was transferred into a new tube, and another 750 μL of extraction buffer was added. The sample was shaken for 3 min and centrifuged at 5,500 g and 4°C for 10 min. Four volumes of precipitation solution (0.1 M ammonium acetate) were gently added onto the upper phenol phase, mixed well, and incubated at -20°C overnight. Following centrifugation at 12,000 g and 4°C for 10 min, the pellet was washed with cold precipitation solution three times and washed once with acetone pre-cooled at -20°C. The supernatant was then discarded after centrifugation, and the tube was opened and dried at -20°C. Following the completion of the freeze-drying process, 500 μL sample hydration solution (8M urea, 1% (W/V) DTT, 2% (W/V) CHAPS, 0.5% (W/V) ampholine) was added into a tube containing 0.05 g protein powder, incubated in a water bath at 28°C for 1 hour with the mixture every 15 min. After centrifugation at 12,000 g and 25°C for 20 min, the supernatant was transferred into a new centrifuge tube, followed by further centrifugation to remove the supernatant. Finally, the protein sample was quantified by the Bradford method and directly used or stored at -80°C.

### 2.5 IMAC

A total of 5 ml of ddH_2_O were added to 1 mL IMAC chromatographic column and thoroughly mixed, and the supernatant was discarded after precipitation on ice (repeated three times). Following this, 2 mL 0.1 M CuSO_4_, ZnSO_4,_ or FeSO_4_ was added to the column and thoroughly mixed, and the supernatant was discarded after precipitation on ice. After the addition of 5 mL ddH_2_O to the column to wash off the unbound metal ions (repeated three times), the sample was washed three times with binding buffer (20 mM NaH_2_PO_4_, 0.5 M NaCl, 0.1% (W/V) Triton X-100). The freshly extracted mitochondria were frozen in liquid nitrogen and thawed repeatedly ten times. After quantification by the Bradford method, the mitochondrial protein sample was diluted to 4 mg/mL with ddH_2_O and then injected into the column. The beads were gently mixed with a pipette and settled down naturally for 10 min. This step was repeated five times, and thus the incubation time used for the binding of the proteins with beads was approximately about 50 min. After washing with binding buffer for four times, the sequential elution was carried out by adding NH_4_Cl with a series of concentration gradients, followed by 50 mM EDTA. The eluents were subsequently detected by SDS-PAGE, Western blot and mass spectrometry.

### 2.6 Liquid chromatograph-mass spectrometry

The samples were digested using the filter-aided sample preparation (FASP) method. More specifically, 200 μg of proteins were incorporated into each sample with 30 μL SDT buffer (4% SDS, 100 mM DTT, 150 mM Tris-HCl, pH 8.0), incubated in boiling water bath for 5 min and cooled to room temperature. Following this, 200 μL UA buffer (8M urea, 150 mM Tris-HCl, pH 8.5) was added to remove the SDS and DTT by repeated ultrafiltration (Microcon units, 10 kD). After the addition of 100 μL iodoacetamide (100 mM IAA in UA buffer) the samples were incubated for 30 min in darkness. This was proceeded by centrifugation at 14,000 g for 15 min. The filters were washed with 100 μL UA buffer three times and then with 100 μL 25 mM NH_4_HCO_3_ buffer twice. Finally, the protein suspensions were digested with 4 μg trypsin (Promega) in 40 μL 25 mM NH_4_HCO_3_ buffer overnight at 37°C, and the resulting peptides were collected as a filtrate. The peptides of each sample were desalted on C18 Cartridges (Empore™ SPE Cartridges C18, bed I.D. 7 mm, volume 3 mL, Sigma, USA), concentrated by vacuum centrifugation and reconstituted in 40 µL of 0.1% (v/v) formic acid. The peptide content was estimated using ultraviolet (UV) light spectral density at 280 nm.

Each fraction was injected into Easy nLC (Thermo Fisher Scientific, USA) for LC analysis. The peptide mixture was loaded onto a reverse phase trap column (Thermo Scientific Acclaim PepMap100, 100 μm*2 cm, nanoViper C18) connected to the C18-reversed phase analytical column (Thermo Scientific Easy Column, 10 cm long, 75 μm inner diameter, 3 μm resin) in buffer A (0.1% Formic acid) and separated with a linear gradient of buffer B (84% acetonitrile and 0.1% Formic acid) at a flow rate of 300 nL/min. The linear one hour gradient is as follows: 0-60% buffer B for 50 min; 60-90% buffer B for 4 min; and hold in 90% buffer B for 6 min.

MS/MS analysis was performed on a Q Exactive mass spectrometer (Thermo Scientific) for one hour in positive ion mode. MS data was acquired using a data-dependent top20 method, dynamically choosing the most abundant precursor ions from the survey scan (300-1800 *m*/*z*) for HCD fragmentation. The automatic gain control (AGC) target was set to 1e6, the maximum inject time to 50 ms, and the number of scan ranges to 1. The dynamic exclusion duration was 30.0 s. Survey scans were acquired at a resolution of 70,000 at 100*m*/*z* and the HCD spectral resolution was set to 17,500 at *m*/*z* 100. Furthermore, the AGC target was set to 1e^5^, the isolation width to 1.5 *m*/*z*, the microscans to 1, and the maximum inject time to 50 ms. The normalized collision energy was 27 eV and the underfill ratio was defined as 0.1%. The instrument was run with the peptide recognition mode enabled.

A database search was performed using the MASCOT search engine (version 2.2., Matrix Science Ltd., UK) on the UniProtKB database with the following parameter settings: peptide mass tolerance of 20 ppm; fragment mass tolerance of 0.1 Da, with trypsin as the enzyme; up to two missed cleavages allowed; carbamidomethylation set as fixed modification; oxidation of methionine was allowed as variable modification.

### 2.7 Western blot and protein carbonylation detection

SDS-PAGE and membrane transfer were performed using the Bio-Rad mini-protean protein electrophoresis system with standard procedures. For Western blot, the polyvinylidene fluoride (PVDF) membrane was washed with TBST (20 mM Tris-HCl, 150 mM NaCl, 0.05% (V/V) Tween 20, pH 8.0) for three times, 5 min each time. For carbonylation detection, the PVDF membrane was immersed in 1×TBS (20 mM Tris-HCl, 150 mM NaCl, pH 7.6) with 20% (W/V) methanol for 5 min to reach an equilibrium, and washed with 2 M HCl for 5 min. Next, the membrane was incubated in 1×DNPH (Cell Biolabs, USA) for 5 min and rinsed with 2 M HCl for three times, 5 min each time. Then it was washed with 50% (W/V) methanol five times, 5 min each time.

Next, a total of 5% (W/V) skimmed milk was added to the membrane and blocked for 1 hour. The primary antibody was diluted with blocking solution (1:2000-1:5000) and incubated at 4°C overnight. The secondary antibody was diluted with blocking solution (1:5000, BioDee, China) and cultured at 25°C for 1 hour. Finally, the protein bands were detected *via* ECL (Millipore, USA). The primary antibodies used in the Western blot were anti-VDAC (cat#AP17092c, Abgent, USA), anti-GFP (cat#GNI4110-GP, GNI, Japan), anti-GST (cat#GNI4110-GT, GNI, Japan) and anti-His antibody (cat#bsm-33004M, Bioss, China). The protein carbonyl groups were detected by anti-DNP antibody in an immunoblot kit (OxiSelect™ ProteinCarbonyl Immunoblot Kit, CELL BIOLABS, INC).

To detect carbonylation of recombinant proteins induced by metal-catalyzed oxidation (MCO), the wild-type or His-mutant UpVDAC protein samples were diluted to 1 μg/μL. Different concentrations of CuSO_4_ and H_2_O_2_ were added and reacted at 25°C in the dark for 30 min. After quantification, 10 μg protein of each sample was used for carbonylation detection as the methods described above.

### 2.8 Site-directed mutation and protein expression

The pMD19-UpVDAC vector was used as the template, and different primersand *Pfu* DNA polymerase were used for the PCR reaction (**Table S1**). The vector with the first site mutation was used as the template when constructing the vector with two site mutations. The point mutation UpVDAC sequence was ligated into the pGEX-4T-1 vector after sequencing. The pGEX-UpVDAC vector was transformed into the *E. coli* BL21 (DE3) strain (Tiangen Biotechnology, China) and induced with 0.5 mM IPTG at 20°C and 140 rpm. The glutathione agarose column (Solarbio, China) was used for protein purification.

### 2.9 Subcellular localization analysis

Tobacco leaves were transfected with Agrobacterium carrying wild-type or point mutant pBI121-UpVDAC-GFP and cultured at 25°C for 2-3 days. The separation buffer was prepared with 125 mM CaCl_2_, 5 mM KCl, 20 mM MES, and 9 g/L NaCl. The leaves expressing GFP were put into a conical flask filled with enzymolysis solution (separation buffer containing 10 g/L cellulose and 2.5 g/L pectinase) and incubated at 28°C at 80 rpm for 4-5 hours. The sample was filtered with an 80 mesh cell sieve, and centrifuged at 600 rpm for 5 min. The supernatant was then discarded and the sample was resuspended by separation buffer followed by two cycles of centrifugation. After staining with 0.5 μg/mL MitoTracker^®^ Red CMXRos (Yisheng Bio, China) for 10 min, the protoplast was washed with PBS three times and observed through a confocal microscope (Leica TCS-SP8 SR, Germany).

### 2.10 pYES2-UpVDAC construction and yeast transformation

The pGEX-UpVDAC vector was digested with *Eco*R I and *Not* I to construct the pYES2-UpVDAC vector. 1 μg plasmid (pYES2/CT, pYES-UpVDAC), 0.1 mg denatured salmon sperm DNA and 100 μL INVSc1 yeast competent cells (Invitrogen, USA) were added to TE solution (pH 8.0, 50 mM Tris-HCl, 1 mM EDTA) containing 110 mM LiAc and 40% (W/V) PEG-3350. After gentle mixing, the samples were incubated at 30°C for 30 min, and mixed every 10 min. Following this, 88 μL DMSO was added and gently mixed, followed by a heat shock at 42°C for 7 min. The sample was then centrifuged at 14,000 g for 10 seconds, and the supernatant was discarded. The yeast were resuspended with 1 mL TE solution, centrifuged again, and suspended with 50 μL TE solution. Finally, the yeast were spread on the SC-Ura solid screening medium and cultured at 30°C for 3-4 days.

### 2.11 Yeast protein expression

Yeast monoclonal was picked up and cultured in 15 mL SC-Ura screening medium (0.067% (W/V) CSM-4 deficiency, 0.268% (W/V) YNB, 0.8% (W/V) glucose, 0.8% (W/V) agar, 4 mg 100×His, 20 mg 100×Leu, 4 mg 100×Trp) at 200 rpm, 30°C for 12 hours. Then it was cultured in the induction medium at 28°C (2% (W/V) galactose, 1% (W/V) raffinose in SC-Ura screening medium). Next, cells were suspended in lysate solution (50 mM sodium phosphate, pH 7.4, 1mM EDTA, 5% (W/V) glycerol, 1mM PMSF) and diluted to an OD_600_ value ranging from 0.5 to 1.0. Equal-volume glass beads were then added for grinding. After centrifugation at 12,000 g for 10 min, the supernatant was detected by Western blot.

### 2.12 Yeast oxidation treatment

Yeasts were transferred with pYES2/CT or pYES-UpVDAC and induced to express proteins. The cells were then collected by centrifugation and resuspended with 0.9% (W/V) NaCl to reach an OD_600_ of approximately 0.4. The resuspended culture was then subjected to gradient dilution (10^1^, 10^2^, 10^3^, 10^4^ times) and cultured in the induction medium containing metal salts and H_2_O_2_ at 30°C for two days in a dark environment to detect yeast growth under different treatments.

### 2.13 Construction of transgenic plants

The pMD19-H204A-UpVDAC and pMD19-H204A+H219A-UpVDAC were used as templates for the PCR reaction and constructed into a pBI121 vector. Agrobacterium GV3101 competent cells were transformed and screened on YEB solid medium containing rifampicin and kanamycin for positive colonies. Under the background of wild-type Arabidopsis Col, the flower-soaking method was used to perform Agrobacterium infection. The harvested seeds were screened for positive strains on a 1/2 MS medium containing 50 mg/L kanamycin, and the harvested seeds were further planted and screened for three generations. The T3 transgenic plants were identified by Western blot using an anti-GFP antibody.

### 2.14 Statistical analysis

The data were analyzed by the univariate general linear model (ANOVA) using SPSS Statistics (IBM, USA). Duncan’s multiple comparison tests and T-tests were used for the significance analysis. For the Duncan multiple comparison method, different letters represent significant differences. In the T-test, * represents *P*<0.05, and ** represents *P*<0.01.

## 3 Results

### 3.1 Metal-binding proteins are target of carbonylation modification during seed aging

**Figure 1:**
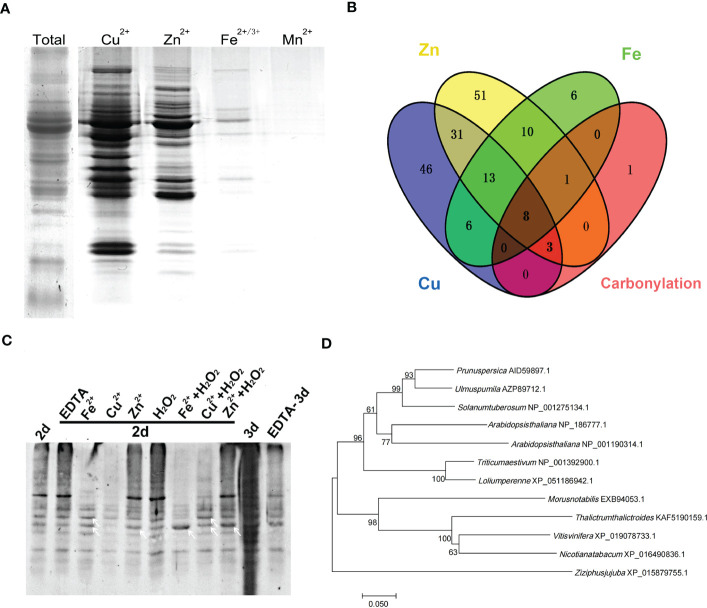
Metal ion binding protein was the target for carbonylation modification during elm seed aging. **(A)** SDS-PAGE analysis of metal-binding proteins in mitochondria of elm seeds captured by immobilized metal affinity chromatography (IMAC). **(B)** Venn diagram of different metal-binding proteins and carbonylated proteins. **(C)** Detection of mitochondrial protein carbonylation in aged elm seeds with different pre-treatments. The elm seeds were pretreated with Fe^2+^ (20 mM), Cu^2+^ (10 mM), Zn^2+^ (30 mM), EDTA (1 μM) or metal salts with H_2_O_2_ (10 mM) for 4 hours. White arrows indicate specific carbonylated protein bands. 2d, seeds aged by controlled deterioration treatment (CDT) for two days; 3d, seeds aged by CDT for three days. **(D)** Phylogenetic tree analysis of VDAC protein in *Ulmus pumila* L. and other species.

Metal-binding proteins are more vulnerable to carbonylation modification (Kryndushkin et al., 2017). To investigate whether metal-binding proteins are the main targets of carbonylation during seed aging, we analyzed the metal-binding proteins in the mitochondria of elm seeds by IMAC. Mitochondrial proteins from non-aged seeds were extracted under non-denaturing conditions, and proteins with metal binding capacity were separated by IMAC columns chelated with Cu^2+^, Zn^2+^, Fe^2+^, and Mn^2+^, respectively, followed by SDS-PAGE detection after elution by the metal chelator EDTA (**Figure 1A**). The results showed that Cu^2+^, Zn^2+^, and Fe^2+^ captured many proteins, while Mn^2+^ barely captured the proteins. The proteins captured by Cu^2+^, Zn^2+^, and Fe ^2+^ were then subjected to LC-MS/MS analysis for identification.

A total of 107 proteins with Cu^2+^ binding capacity, 117 with Zn ^2+^ binding, and 44 with Fe^2+^ binding capacity were identified by mass spectrometry. Excluding intersecting proteins bound to multiple metals, 188 metal-binding proteins were successfully identified (**Tables S2–S4**). Our previous proteome study revealed that 13 proteins were carbonylation modified during the aging of elm seeds (Li et al., 2017). Merging the previous carbonylation proteome data and the new data of the metal-ions captured by proteome reveals 12 carbonylated proteins to be metal-binding proteins detected by IMAC in this study. Only one protein was detected to have an unknown function. Further analysis showed that the majority of these 12 proteins undergoing carbonylation could bind Cu, Zn, or Fe (**Figure 1B**; **Table 1**).

**Table 1:** IMAC of carbonyl mitochondrial protein during elm seed aging.

Protein description	Proteins captured by Cu^2+^		Proteins captured by Zn^2+^		Proteins captured by Fe^2+^		Molecular weight
	Accession number	Cover percent	Accession number	Cover percent	Accession number	Cover percent	
Malate dehydrogenase	W9S9W9	17.48%	W9S9W9	15.76%	W9S9W9	15.76%	36489.57
	G3BMW0	17.99%	G3BMW0	16.22%	G3BMW0	16.22%	35455.44
					W9QNR6	10.86%	41903.67
Heat shock protein, 70 family	W9SKK5	20.63%					71725.46
			R4L6S9	5.50%			71267.73
Heat shock protein, putative	Q8H6U4	22.02%	Q8H6U4	21.65%	Q8H6U4	28.44%	57762.96
	W9SBQ7	11.07%	W9SBQ7	14.72%	W9SBQ74	13.08%	90068.66
Chaperonin-60kD, ch60, putative	W9SB24	30.84%	W9SB24	36.59%	W9SB24	39.55%	61276.96
	W9RE69	24.34%	W9RE69	26.72%	W9RE69	28.57%	40809.06
	W9R5Z3	17.54%	W9R5Z3	21.18%	W9R5Z3	28.70%	48869.50
PREDICTED: mitochondrial-processing peptidase subunit α-like	W9RH22	7.33%	W9RH22	9.11%	W9RH22	7.52%	54235.00
	W9QJ01	5.51%	W9QJ01	5.51%	W9QJ01	7.28%	54946.51
			W9QID6	11.99%	W9QID6	20.22%	59112.28
ATP synthase subunit α, mitochondrial	W9R2H6	20.72%	W9R2H6	30.99%	W9R2H6	36.04%	59321.85
ATP synthase subunit α	B5KXY5	26.26%			B5KXY5	33.04%	49404.35
	B5KXY6				42.27%	50039.00
	J7MPY1				40.20%	55348.82
Mitochondrial outer membrane protein porin of 36 kDa	W9R9M3	5.43%	W9R9M3	25.00%	W9R9M3	22.46%	29435.75
Mitochondrial outer membrane protein porin of 34 kDa	W9QZM5	8.70%	W9QZM5	13.77%	W9QZM5	13.04%	29615.99

Previous studies showed that mitochondrial protein carbonylation modification levels did not changed significantly when elm seeds were aged for two days, although the modification levels were aggravated after three days of aging (Li et al., 2017). To verify the relationship between metal ions and carbonylation modification during seed aging, elm seeds were pretreated with Fe^2+^ (20 mM), Cu^2+^ (10 mM), Zn^2+^ (30 mM), EDTA (1 μM) or metal salts with H_2_O_2_ (10 mM) for two days. The mitochondrial proteins were then extracted and subjected to SDS-PAGE separation followed by carbonylation detection. The results showed aggravation of global protein carbonylation after aging for two days under the Zn^2+^/H_2_O_2_/Zn^2+^+H_2_O_2_ pretreatment. Fe^2+^ or Cu^2+^ even reduced the levels of carbonylation modifications, possibly due to protein degradation by metal oxidation. However, Fe^2+^+H_2_O_2_/Cu^2+^+H_2_O_2_ significantly enhanced carbonylation of some specific proteins (indicated by white arrows) compared with metal-ion treated sample, suggesting a result of metal-catalyzed oxidation (MCO). When EDTA treatment was applied to the seeds, followed by aging for three days, the aggravated carbonylation was reversed (**Figure 1C**). The effect of EDTA was not observed when pretreatments were applied to seeds aged for two days, at which the carbonylation was not overloaded to cells compared with CDT-3d.

### 3.2 Site-directed mutagenesis and purification of VDAC protein

Notably, among the proteins captured by IMAC, VDAC is a protein with the binding capacity for all three metals (**Tables S2–S4**). Research has demonstrated that VDAC carbonylation is aggravated during aging, and metal salt treatment can cause changes in the VDAC channel function by *in vitro* experiments with mitochondria (Li et al., 2017). In the current study, the phylogenetic tree of VDAC was analyzed using the bootstrap consensus tree classification tool in the bioinformatics software MEGA 7.0 (Mega Limited, New Zealand) (**Figure 1D**). UpVDAC presented a high homology with the VDACs of several other higher plants, and exhibited the highest homology with that of *Prunus persica* up to (87.68%). Among the three proteins VDAC1, 2 and 3 in Arabidopisis (NP_186777.1, NP_001318898.1, NP_001190314.1), UpVDAC had 72.46% homology with AtVDAC1 and 68.48% homology with AtVDAC3.

**Figure 2:**
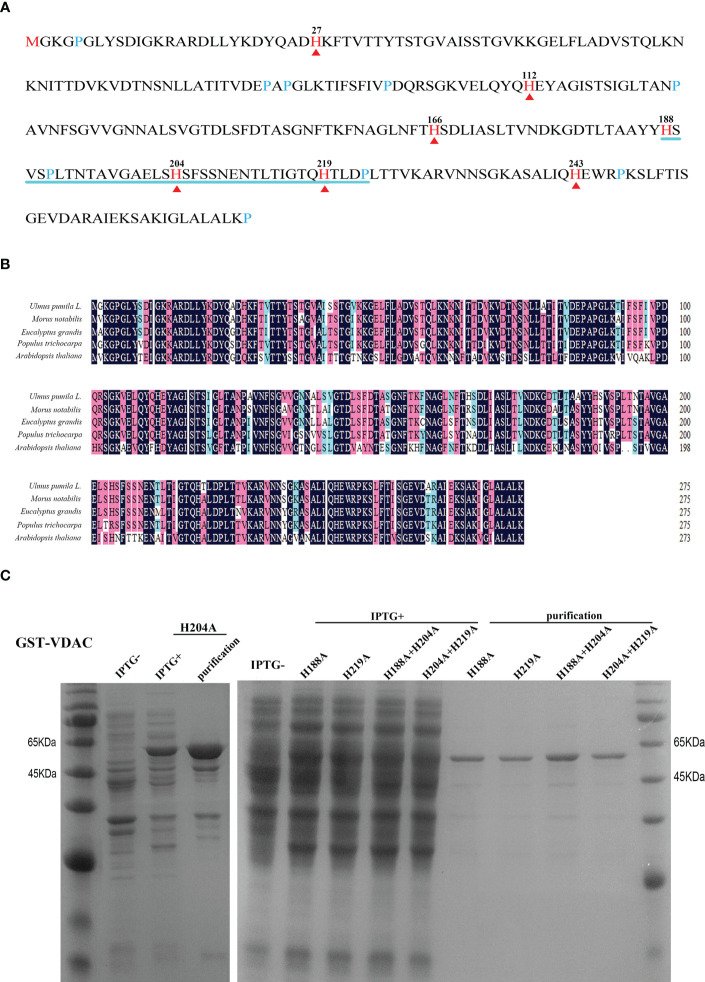
Amino acid sequence analysis, expression, and purification of VDAC protein. **(A)** Sequence analysis of the UpVDAC protein. Red arrowheads and numbers indicate seven histidine (His, H) residues. Blue lines are drawn under three adjacent His. **(B)** Alignment of VDAC homologous protein sequences from different species. **(C)** Prokaryotic expression and purification of wild type and site-directed mutated GST-UpVDAC recombinant proteins: H188A; H219A; H188A+H204A; H204A +H219A.

To explore the role of VDAC carbonylation in seed aging, we first explored the possible association of VDAC carbonylation modification with the metal binding ability. Previous studies have found that His residues in a protein may serve as the metal binding and oxidation-susceptible sites (Tan et al., 2010). VDAC has seven His residues (**Figures 2A, B**), among which the 4^th^ (H188) and 6^th^ (H219) His are adjacent to another oxidation-susceptible residue (Pro). The distance of 5^th^ His (H204) to H188 (15 amino acid residues) or to H219 (14 amino acid residues) on the peptide chain is basically equal. To explore whether these three His were metal binding and MCO targeted sites, site-directed mutagenesis was performed with one or two His mutated to alanine (Ala, A). The mutated proteins H188A, H204A, H219A, H188A+H204A, and H204A+H219A were purified by prokaryotic expression to obtain soluble expressed proteins (**Figure 2C**).

### 3.3 Metal-binding ability and carbonylation modification sites of VDAC

**Figure 3:**
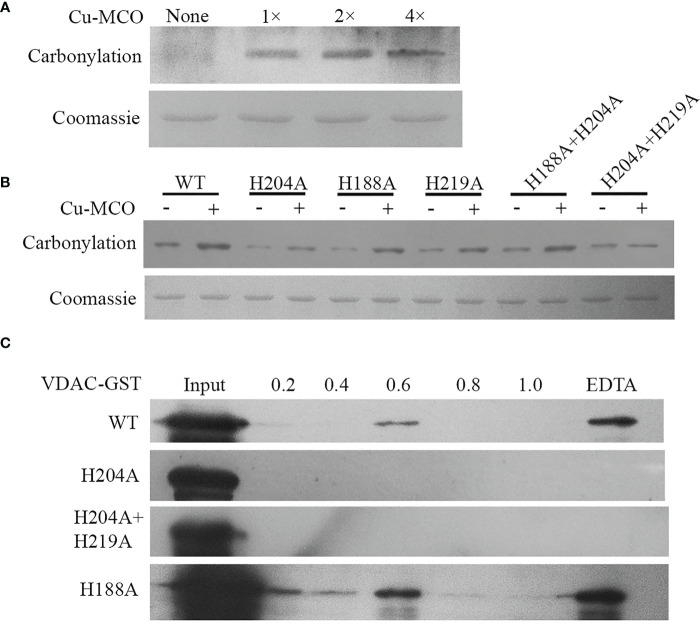
Carbonylation detection and metal binding ability of wild type and site-directed mutated GST-UpVDAC recombinant proteins. **(A)** Detection of carbonylation modification of WT-UpVDAC with different treatments: 1×, 0.2 mM CuSO_4 + _0.5 mM H_2_O_2_; 2×, 0.4 mM CuSO_4 + _1 mM H_2_O_2_; 4×, 0.8 mM CuSO_4 + _2 mM H_2_O_2_. Cu-MCO, Cu+H_2_O_2_ treatment. **(B)** Detection of carbonylation of UpVDAC proteins with different His site mutations after treatment with 2× CuSO_4_ and H_2_O_2_. WT, wild-type UpVDAC protein. **(C)** IMAC analysis of the metal binding capacity of WT and His site mutant UpVDAC. Column-trapped proteins were eluted with a series of NH_4_Cl concentrations and the final elution with EDTA, followed by Western blot detection.

To verify the effect of His site mutations on the sensitivity of protein to carbonylation modification, the sensitivity of wild type (WT)/His site mutated UpVDAC protein to metal-initiated carbonylation modification was analyzed. First, CuSO_4_ and H_2_O_2_ treatment gradient concentrations were applied to prokaryotic-expressed WT-UpVDAC. Following this, using the DNPH carbonyl reaction and anti-DNPH antibodies, the levels of protein carbonylation modification were examined under different treatments. As shown in **Figure 3A**, a minimal carbonylation signal was detected for UpVDAC protein without metal treatment, while treatment with 0.2 mM CuSO_4_ and 0.5 mM H_2_O_2_ (1×) significantly aggravated its carbonylation. The UpVDAC carbonylation increased when the CuSO4 and H_2_O_2_ concentrations were doubled (2×), and it remained when the CuSO_4_ and H_2_O_2_ concentrations continued to increase (4×). Therefore, 0.4 mM CuSO_4_ and 1 mM H_2_O_2_ (2×) were selected for the subsequent experiments.

The carbonylation modification of WT/His site mutated UpVDAC proteins was tested with the 2× oxidative treatment, and the proteins without treatment were used as controls. The results showed that H204A+H219A lost sensitivity to metal-initiated carbonylation modification, while H204A showed reduced sensitivity (**Figure 3B**). However, other His mutations have little effect on the carbonylation modification of UpVDAC.

Then we analyzed the metal binding ability of the UpVDAC protein using IMAC and Western blot assay. The purified WT-UpVDAC, H188A as well as mutants with reduced sensitivity to metal-induced carbonylation H204A and H219A+H204A were incubated with an IMAC-Cu column, respectively. After washing away unbound protein with PBS and elution with gradient NH_4_Cl, we found that for the WT-UpVDAC protein, only the higher concentration (0.6 μM) NH_4_Cl could partially elute the bound protein (**Figure 3C**), and the rest of the more tightly bound protein could only be finally eluted by EDTA, demonstrating the strong Cu binding ability of UpVDAC. However, H204A or H204A+H219A protein was not detected in each concentration of the NH_4_Cl and EDTA elutes but washed out at the PBS step, indicating that the mutant protein had lost its ability to bind Cu. H188A, the mutant protein sensitivity to carbonylation, was eluted by NH_4_Cl and EDTA, demonstrating that mutation at this site did not affect the metal binding ability of the UpVDAC protein (**Figure 3C**).

To verify whether the His site mutation affected UpVDAC localization in mitochondria, eukaryotic expression vectors for WT and His mutated UpVDAC-GFP were constructed and transiently expressed in tobacco. Mitochondria were labeled using the Mito-tracker, a mitochondrial fluorescent probe. The result of confocal microscope showed that the His mutations H204A and H204A+H219A did not alter the UpVDAC localization in mitochondria (**Figure S1**).

### 3.4 Effects of VDAC carbonylation in yeast cells under oxidative stress

**Figure 4:**
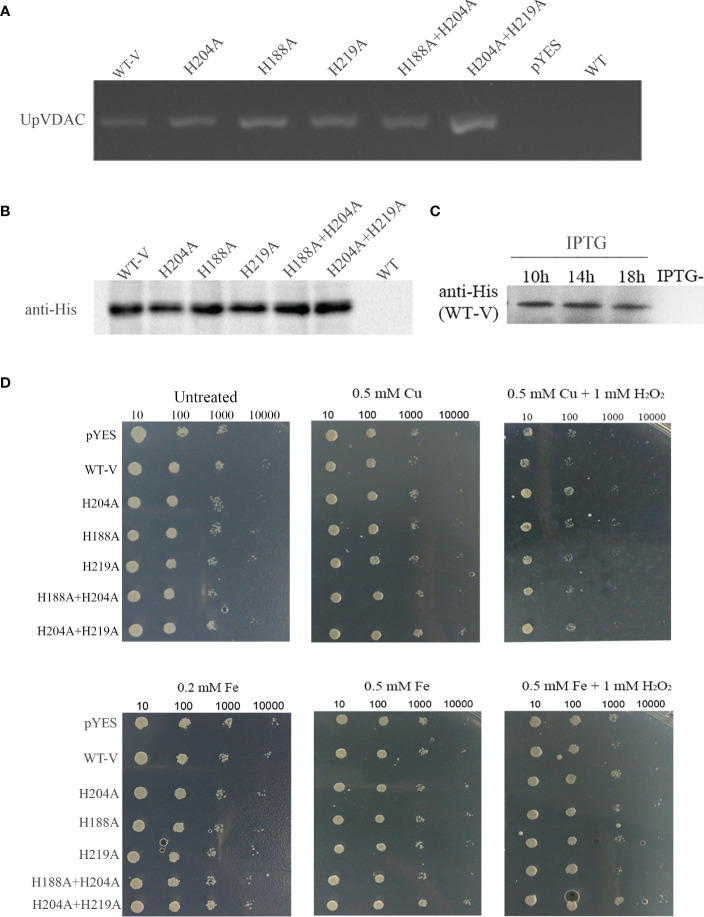
Growth status of yeast overexpressing WT/His-site mutated-UpVDAC under metal salt treatment. **(A)** Confirmation of yeast transformants by PCR. **(B)** Protein expression of yeast transformants detected by Western blot. **(C)** Proteins expression of yeast transformants at different induction times determined by Western blot. **(D)** Growth status of yeast strains under Cu^2+^/Cu^2+^+H_2_O_2_ (Cu-MCO)/Fe^2+^/Fe^2+^+H_2_O_2_ (Cu-MCO) treatment with indicated concentrations. pYES, yeast overexpressing pYES2 empty vector; WT-V, yeast overexpressing wild-type UpVDAC protein.

The physiological function of VDAC carbonylation was subsequently investigated in yeast. The WT/His-mutated UpVDAC yeast expression vectors were transferred into the INVSc1 yeast strain, and the transformants were confirmed by PCR (**Figure 4A**). Following this, the yeast was subjected to inducible expression, and the UpVDAC protein expression was detected by Western blot. The results showed that all the yeast stains could transiently express the WT/His-mutated UpVDAC protein normally (**Figure 4B**). The amount of UpVDAC protein in yeast was almost constant after a 10-hour induction (**Figure 4C**). Thus, the induction time was set as 10 hours for the subsequent experiments.

To verify the relationship between metal and VDAC carbonylation for cell vitality, yeast with an overexpression of WT-UpVDAC or with His site mutations was treated with Fe^2+^, Cu^2+^ or metal salts with H_2_O_2_. The yeast strain transformed with the pYES2/CT empty vector was used as a control. Compared to the untreated yeast, various yeast strains showed similar growth status under the Fe^2+^or Cu^2+^ treatments. However, the growth was obviously inhibited with 0.5 M Cu+1 mM H_2_O_2_ (Cu-MCO) or 0.5 M Fe+1 mM H_2_O_2_ (Fe-MCO)-induced oxidative stress (**Figure 4D**), indicating that treatment with metal salt and H_2_O_2_ could reduce yeast viability. Compared with yeast overexpressing pYES2 empty vector (pYES), yeast with an overexpression of WT-UpVDAC (WT-V) was more sensitive under the treatment of Cu-MCO or Fe-MCO. This was indicated by sparser colonies grown out from 1:100 dilution under Cu-MCO, and sparser colonies from 1:1000 dilution under Fe-MCO. When the mutation protein insensitivity to MCO-induced carbonylation UpVDAC H204A or H204A+H219A was overexpressed, the growth potential under MCO treatment was better than WT-V. When other mutation was overexpressed, the growth potential of yeast was similar to the control under Cu-MCO or between WT-V and MCO insensitivity mutants under Fe-MCO. The above results illustrate that VDAC overexpression leads to a more sensitive phenotype of yeast cells to oxidative stress, and carbonylation site mutations can abolish the effects of VDAC overexpression.

### 3.5 Role of UpVDAC carbonylation modification in Arabidopsis seedling growth and seed aging

**Figure 5:**
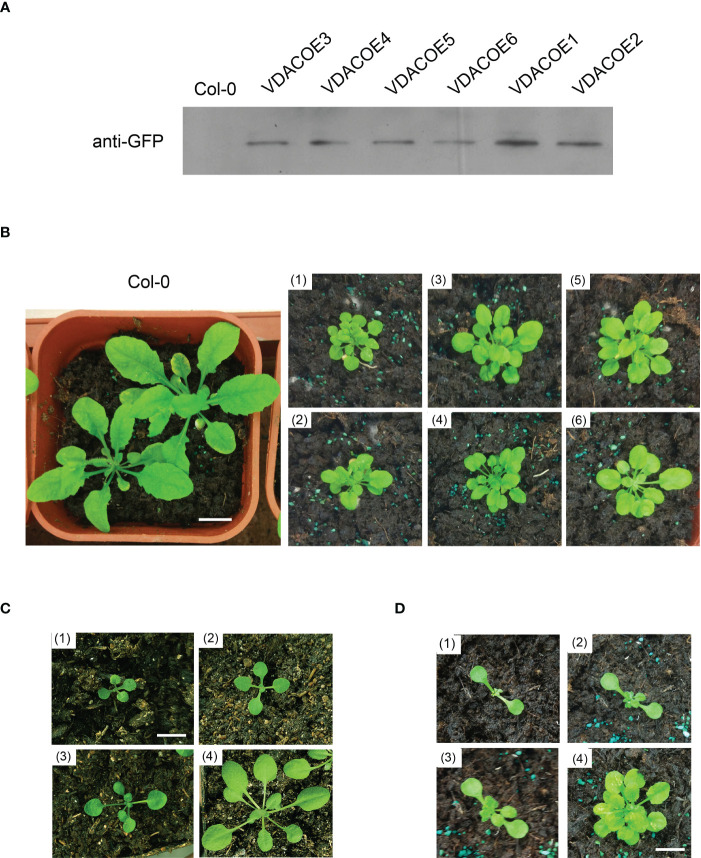
Phenotype of Arabidopsis seedlings overexpressing wild-type (WT) UpVDAC. **(A)** Confirmation of transgenic lines by Western blot with anti-GFP antibody. **(B)** Seedling phenotype of several transgenic Arabidopsis strains overexpressing WT UpVDAC. Col-0, WT Arabidopsis ecotype Columbia; (1) - (6), seedling phenotypes of VDACOE1-VDACOE6; scale bar = 1 cm. **(C)** Seedling phenotypes of WT Arabidopsis at four different growth periods. Col-0, WT Arabidopsis ecotype Columbia; (1) - (4), seedling of 10-30 days after germination; scale bar = 1 cm. **(D)** Seedling phenotypes of Arabidopsis stain overexpressing WT-UpVDACVDACOE1 at four different growth periods. (1) - (4), seedling of 10-30 days after germination; scale bar = 1 cm.

To investigate the physiological role of UpVDAC and its carbonylation modification in plants, the T3 generation of Arabidopsis strains overexpressing WT or the MCO insensitivity UpVDAC mutants were obtained. Proteins were extracted from the seeds of different lines, and UpVDAC expression was detected using antibodies against GFP. **Figure 5A** shows that UpVDAC-GFP proteins were successfully expressed in transgenic Arabidopsis strains VDACOE1-6. Phenotypic comparisons were made between different transgenic plant lines. Compared with the wild-type control, six WT-UpVDAC over-expressed lines showed retarded growth of seedlings (**Figure 5B**). Taking VDACOE1 as an example, during different growth periods, VDACOE lines exhibited a lag in real leaf growth and clustered rosettes (**Figures 5C, D**). These phenotypes suggest that UpVDAC plays an essential role during seedling morphogenesis.

**Figure 6:**
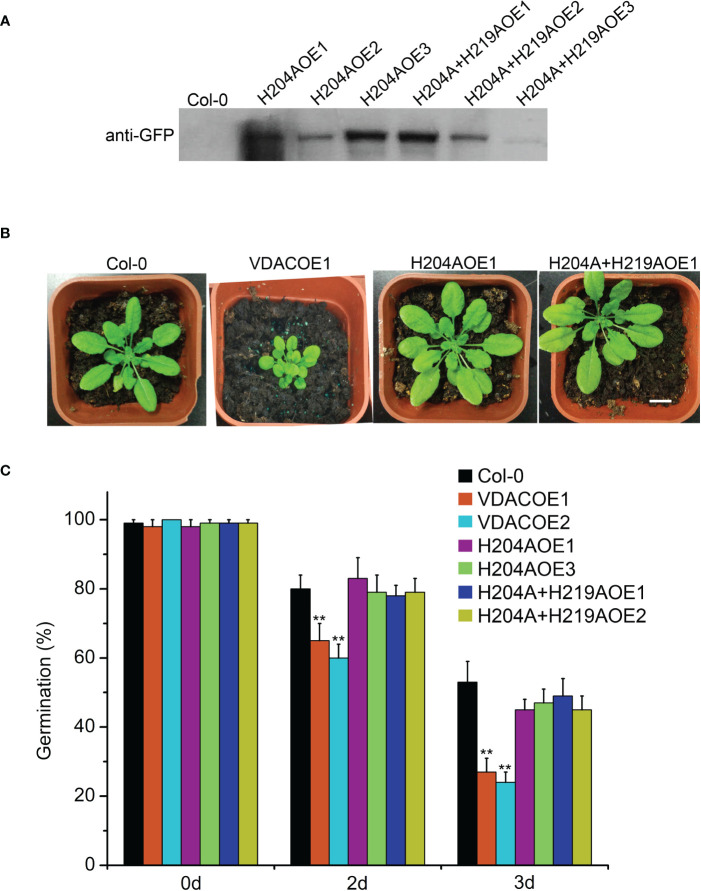
Phenotype of Arabidopsis seedlings and seeds overexpressing wild-type (WT) or metal-binding site mutated UpVDAC. **(A)** Confirmation of transgenic lines by Western blot with anti-GFP antibody. **(B)** Phenotypic comparison between Arabidopsis seedlings overexpressing WT or metal binding site mutant UpVDAC. Col-0, WT Arabidopsis ecotype Columbia. Scale bar = 1 cm. **(C)** Germination percentages of different Arabidopsis seeds after CDT triggered aging. Col-0, WT Arabidopsis ecotype Columbia. Significance analysis was performed using the *t*-test. ^**^ *P* < 0.01.

The transgenic Arabidopsis plants of MCO-insensitivity UpVDAC mutants were obtained and confirmed by Western blot. Among the three H204A transgenic lines, H204AOE1 showed the highest expression level. H204A+H219AOE1 exhibited higher levels than the other two lines (**Figure 6A**). For each mutated protein, phenotypic analysis was performed in two transgenic lines with higher VDAC expression, H204AOE1 and H204AOE3, as well as H204A+H219AOE1 and H204A+H219AOE2. Interestingly, H204AOE1 and H204A+H219AOE1 seedlings were indistinguishable from the wild-type in terms of the growth status (**Figure 6B**). This implies that H204 in UpVDAC strongly contributed to the function of UpVDAC, which may be related to the metal ion binding/carbonylation of this residue.

Arabidopsis seeds from different transgenic lines were subjected to CDT to detect seed vigor. This shows that the germination percentage of VDACOE1 and VDACOE2 was significantly lower than that of the wild-type seeds after aging for two or three days, indicating that the overexpression of wild-type UpVDAC accelerated seed aging. However, the germination percentages of metal-binding site mutated VDAC transgenic seeds, H204AOE1/3 or H204A+H219AOE1/2, were not significantly different from that of wild-type seeds after aging (**Figure 6C**). This demonstrates that metal-triggered carbonylation modification exerts a substantial regulatory effect on the function of VDAC in CDT-triggered seed aging.

## 4 Discussion

Carbonylation modification is a key result of protein oxidative damage. The carbonylation modification of specific proteins occurs under aging and other oxidative stress conditions. Amino acids such as Arg, Lys, Pro, and His may be the main sites of carbonylation modification. IMAC and mass spectrometry analysis revealed that proteins with a metal binding ability constitute a significant group of carbonylated modifications (Maisonneuve et al., 2009). The redox active metal can undergo a Fenton reaction with H_2_O_2_ to generate highly oxidizing OH ions, causing strong oxidative stress (Sharma and Dietz, 2009). Metal ions induced protein oxidative damage or metal-catalyzed protein oxidation, leading to the carbonyl modification of proteins. Previous research demonstrated that free metal maybe released during aging and mitochondrial proteins undergo selective carbonylation modification, yet how these two events are related remains unclear (Li et al., 2017). IMAC, combined with protein spectrum identification, reveals that the proteins with increased carbonylation modification in mitochondria during aging are almost metal-binding proteins that can bind Fe, Cu, or Zn (**Figure 1**; **Table 1**). In addition, metal salt combined with H_2_O_2_ pretreatment aggravated the carbonylation of specific mitochondrial proteins after two days of aging. Based on these results, it is speculated that metal-binding proteins may be a major target for protein carbonylation during seed aging.

The electron donor dense region on the protein surface can bind metal ions, and in the presence of ROS, the metal-bound amino acid residues are easier to be oxidized (Stadtman, 1993). Tandem mass spectrometry analysis of the peptide has shown that the amino acid site near the Cu binding site is prone to oxidative modification (Bridgewater et al., 2006). The metal binding site of aconitase is very close to the carbonylation modification site under metal stress after folding into a higher protein structure. Thus, it is speculated that the metal binding ability of the protein may be related to carbonylation modification (Tan et al., 2010). More than 400 endogenous carbonylation modification proteins and over 20 endogenous modification sites have been successfully identified during ferroptosis, and carbonylation modification sites on VDAC2 have been explored (Chen et al., 2018). Our IMAC results show that UpVDAC protein has three metal binding abilities (**Tables S2–S4**), which may explain why UpVDAC becomes the target of carbonylation modification during aging. The His mutation at positions 204 and 219 makes UpVDAC lose its metal binding ability, indicating that these two His sites may be the main metal binding sites of UpVDAC (**Figure 3C**). His mutation also reduced the sensitivity of UpVDAC to metal-induced carbonylation, indicating that metal binding is closely related to UpVDAC carbonylation.

As the main channel protein in the outer membrane of mitochondria, VDAC plays a role in the transport of metabolites between mitochondria and cytoplasm, and functions in the release of mitochondrial pro-apoptotic factors during apoptosis. There are many ways to regulate the channel function of VDAC, including forming protein polymers to change the channel aperture (Zalk et al., 2005). Numerous studies have found that carbonylation can cause protein aggregation, thus affecting protein functions. Aggregated proteins due to carbonyl modification are challenging to degrade (Dalle-Donne et al., 2003). Yeast growth experiments showed that WT UpVDAC could exacerbate the decrease of yeast cell viability under oxidative stress, while the metal-binding site mutations could not (**Figure 4D**). Assays in Arabidopsis protoplasts revealed that the metal-binding site mutation did not affect the subcellular localization of UpVDAC on mitochondrion (**Figure S1**). As the reduced carbonyl modification of metal-binding site mutant proteins under MCO (**Figure 3B**), it is speculated that carbonyl modification of UpVDAC might lead to reduction in cell viability under oxidative stress.

Previous studies have identified ROS-induced PCD as a potentially key reason for seed aging (El-Maarouf-Bouteau et al., 2011; Hu et al., 2012). The mitochondrial morphological and functional changes observed with the increase in ROS are early events in seed aging (Wang et al., 2015). This implies that the cell death type in seed aging is the PCD of the mitochondrial pathway. Past research has found that functional changes in VDAC channels caused by carbonylation modifications may play an important role in cell death *via* the mitochondrial pathway during seed aging (Wang et al., 2015; Li et al., 2017). A study on the fruit aging mechanism reveled that an increase in the carbonylation levels of VDAC with aging induced changes in the mitochondrial membrane permeability (Qin et al., 2009). This indicates that VDAC carbonylation plays an important role in the aging process. In the current study, the overexpression of wild-type UpVDAC accelerated the seed aging of Arabidopsis (**Figure 6C**). When the metal binding sites were mutated, the role of UpVDAC in accelerating aging was alleviated, demonstrating that the metal binding sites of UpVDAC played an important role in accelerating aging. Our results preliminarily proved the function of the VDAC carbonylation in the regulation of cell vitality, seedling growth and seed aging. During seed aging, the destruction of metal-binding proteins may increase the free-metal content of cells, causing further outbreak of ROS. On the other hand, ROS may promote the carbonylation of VDAC, which is likely to affect the function of VDAC in PCD and ultimately lead to a decrease in seed vitality.

## Data availability statement

The original contributions presented in the study are included in the article/**Supplementary Material**. Further inquiries can be directed to the corresponding author.

## Author contributions

YL, HX and XW designed the research. YL, YW and TY performed the experiments and data analysis. CL, MQ and YK wrote the manuscript. HX, YL and TY revised the manuscript. All authors contributed to the article and approved the submitted version.
