# Supplementary material for: Effect of the metal ion-induced carbonylation modification of mitochondrial membrane channel protein VDAC on cell vitality, seedling growth and seed aging
Source: Front Plant Sci. 2023 May 31;14:1138781. doi: 10.3389/fpls.2023.1138781 (PMC10264620; doi:10.3389/fpls.2023.1138781)
Supplement: Supplementary file 1 [file DataSheet_1.docx]

Supplementary Material

Effect of metal ions-induced carbonylation modification of mitochondrial membrane channel protein VDAC on cell vitality and seed aging

**Ying Li†, Chang Liu†, Manyao Qi†, Tiantian Ye†, Ying Kang, Yu Wang, Xiaofeng Wang, HuaXue***

*** Correspondence:** HuaXue: xuehua2013@bjfu.edu.cn

# Supplementary Figures and Tables

## Supplementary Figures


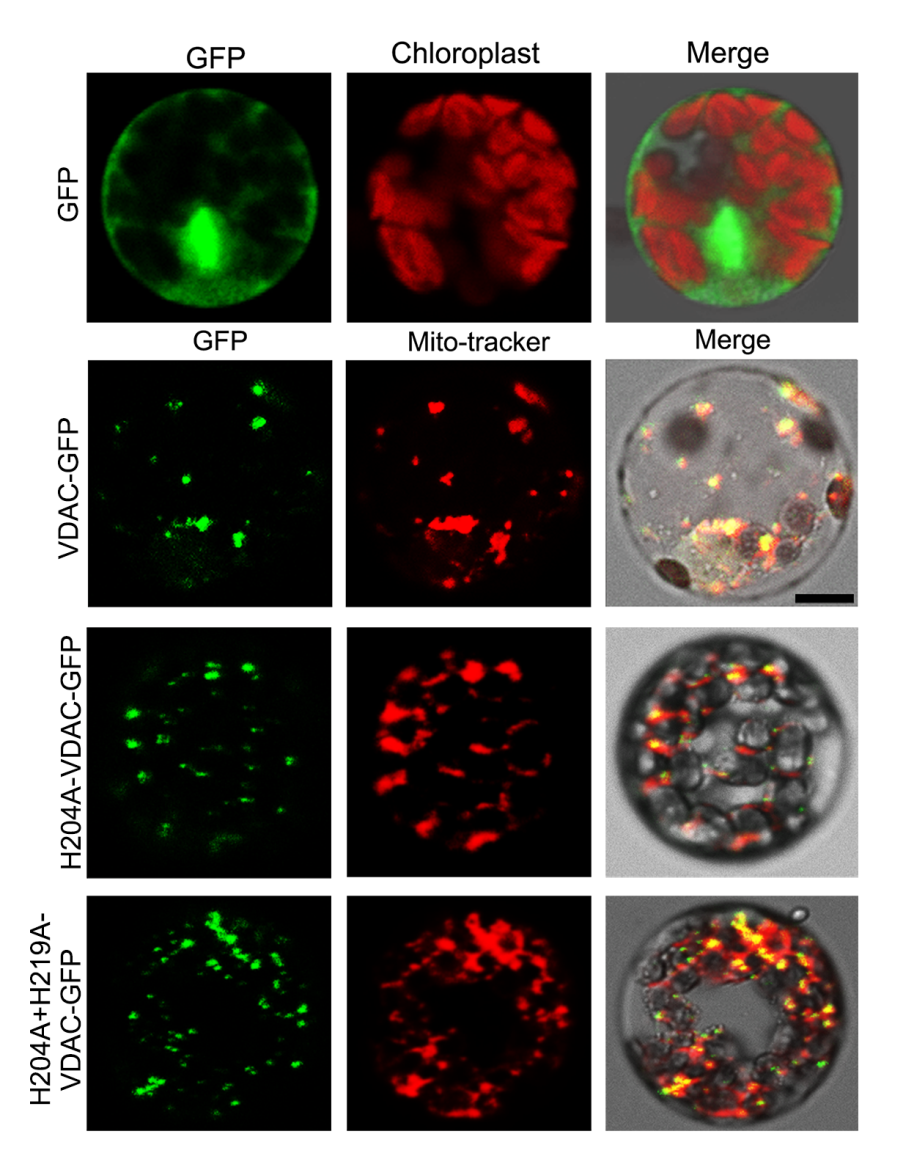


**Supplementary Figure 1.** Subcellular localization of WT and His mutated UpVDAC. Mito-tracker is a mitochondrial fluorescent probe. Scale bar=14 μm.

## Supplementary Tables

**Table S1 The list of primers used in PCR.**

| **Vector** | **Inserted gene** | **Primers** |
| --- | --- | --- |
| pET32a | UpVDAC3 | 5’-CGGAATTCATGGGAAAAGGCCCAGGTCTCT-3’ (*EcoR* I)  5’-TTGCGGCCGCAGGCTTGAGAGCCAAGGCTAGA-3’ (*Not I*) |
| pBI121 | H204A-UpVDAC3 | 5’-GCTCTAGAATGGGAAAAGGCCCAGGTCTCT-3’ (*Xba* I)  5’-CGGGATCCAGGCTTGAGAGCCAAGGCTAGA-3’ (*Bam*H I) |
|  | H204A+H219A-UpVDAC3 | 5’-GCTCTAGAATGGGAAAAGGCCCAGGTCTCT-3’ (*Xba* I)  5’-CGGGATCCAGGCTTGAGAGCCAAGGCTAGA-3’ (*Bam*H I) |
| pMD19-T | H188-UpVDAC3 | 5’-CTTACCGCCGCCTACTACGCGTCTGTCAGCCC -3’  5’- CGCGTAGTAGGCGGCGGTAAGGGTATCACCCT -3’ |
|  | H204-UpVDAC3 | 5’- GTTGGTGCGGAGTTATCCGCCAGCTTCTCAA -3’  5’- GCGGATAACTCCGCACCAACAGCTGTGTTGG -3’ |
|  | H219-UpVDAC3 | 5’- CTCACAATCGGCACCCAAGCGACACTGGATCC -3’  5’-CGCTTGGGTGCCGATTGTGAGTGTGTTTTCGT -3’ |
|  | H188+H204-UpVDAC3 | 5’- CTTACCGCCGCCTACTACGCGTCTGTCAGCCC -3’  5’- CGCGTAGTAGGCGGCGGTAAGGGTATCACCCT -3’  (Cloning with pMD19-H204A-UpVDAC3 as a template) |
|  | H204+H219-UpVDAC3 | 5’- CTCACAATCGGCACCCAAGCGACACTGGATCC -3’  5’- CGCTTGGGTGCCGATTGTGAGTGTGTTTTCGT -3’  (Cloning with pMD19-H204A-UpVDAC3 as the template) |

**Table S2 Cu^2+^ -IMAC captured metal-binding proteins detected by LC-MS/MS.**

| **Description** | **Accession number** | **UniquePepCount** | **MW** | **pI** |  |
| --- | --- | --- | --- | --- | --- |
| ATP synthase F1 subunit 1 | A0A0M5M1Z3 | 18 | 55322.9 | 5.84 |  |
| Uncharacterized protein | M5XYE7 | 18 | 61197.76 | 5.62 |  |
| Chaperonin CPN60-2 | W9SB24 | 17 | 61276.96 | 5.65 |  |
| ATP synthase subunit alpha (Fragment) | A0A0B4L1A3 | 15 | 54966.25 | 5.84 |  |
| Heat shock 70 kDa protein | W9SKK5 | 15 | 71725.46 | 5.94 |  |
| Heat shock protein 60 (Fragment) | Q8H6U4 | 13 | 57762.96 | 5.26 |  |
| Uncharacterized protein | M5VP31 | 13 | 79504.69 | 8.6 |  |
| Aconitatehydratase | W9RLC6 | 12 | 102299.8 | 8.07 |  |
| ADP,ATP carrier protein 3 | W9QNM9 | 11 | 40398.07 | 9.7 |  |
| Aldehyde dehydrogenase family 2 member | W9RMT5 | 10 | 59392.13 | 8.3 |  |
| ATP synthase subunit beta | W9R2H6 | 9 | 59321.85 | 5.83 |  |
| Mitochondrial adenine nucleotide translocator | A0A0D5W7G4 | 9 | 41887.66 | 9.77 |  |
| Succinyl-CoA ligase subunit beta | W9R0G6 | 9 | 45263.87 | 6.07 |  |
| Luminal-binding protein | W9RWI9 | 9 | 73496.4 | 5.04 |  |
| ER-binding protein | A9UKE0 | 8 | 73570.37 | 5.14 |  |
| ATP-dependent zinc metalloprotease FTSH 10 | W9RUG9 | 8 | 90641.94 | 6.98 |  |
| NADH dehydrogenase subunit 7 (Fragment) | A0A0B4L0T7 | 8 | 44542.39 | 6.71 |  |
| 2-oxoglutarate dehydrogenase | W9R2F3 | 8 | 115922.2 | 6.62 |  |
| Heat shock protein 90 | W9SBQ7 | 8 | 90068.66 | 5.16 |  |
| Arginase | B3F0K4 | 7 | 37000.83 | 6.14 |  |
| Dihydrolipoyl dehydrogenase | W9R882 | 7 | 52744.14 | 6.9 |  |
| NADH dehydrogenase [ubiquinone] iron-sulfur protein 1 | W9RU22 | 7 | 81677.63 | 6.64 |  |
| Pyruvate dehydrogenase E1 component subunit alpha | W9RNK6 | 6 | 35897.7 | 7.11 |  |
| Malate dehydrogenase | G3BMW0 | 6 | 35455.44 | 8.98 |  |
| Formate dehydrogenase | W9SX88 | 6 | 42196.64 | 6.68 |  |
| Elongation factor 1-alpha | G8H5D3 | 6 | 49297.49 | 9.15 |  |
| ATP synthase subunit d | W9SNX9 | 6 | 19717.04 | 5.1 |  |
| Succinate dehydrogenase [ubiquinone] flavoprotein subunit, mitochondrial | W9SD93 | 5 | 68781.74 | 6.46 |  |
| NADH dehydrogenase subunit 9 | A0A0M4R4N3 | 5 | 22667.22 | 6.53 |  |
| Glycerol-3-phosphate dehydrogenase | W9RLH6 | 5 | 68631.83 | 8.66 |  |
| Delta-1-pyrroline-5-carboxylate dehydrogenase 12A1 | W9QIA3 | 4 | 71852.25 | 7.65 |  |
| Nucleoside diphosphate kinase | M5WAF8 | 4 | 25804.14 | 9.18 |  |
| Dihydrolipoyl dehydrogenase | M5XYW7 | 4 | 54125.37 | 6.41 |  |
| NADH dehydrogenase [ubiquinone] iron-sulfur protein 7 | W9RE37 | 4 | 23555.12 | 9.63 |  |
| Mitochondrial-processing peptidase subunit alpha | W9RH22 | 4 | 54235 | 6.1 |  |
| Cytochrome c oxidase subunit 2 | F8T950 | 4 | 28916.11 | 5.4 |  |
| Atp4 protein | J7MB97 | 4 | 22088.73 | 9.5 |  |
| Serine hydroxymethyltransferase | M5VXS4 | 4 | 57050.54 | 8.39 |  |
| Glutamate dehydrogenase | M5WMA0 | 4 | 44894.98 | 6.19 |  |
| Glyceraldehyde-3-phosphate dehydrogenase (Fragment) | W8VA63 | 4 | 36179.04 | 8.28 |  |
| Malate synthase | W9RKE6 | 4 | 65002.55 | 8.28 |  |
| Fumaratehydratase 1 | W9RTY2 | 4 | 53269.23 | 6.84 |  |
| Mitochondrial uncoupling protein 3 | W9S5K1 | 4 | 39064.66 | 9.49 |  |
| Dihydrolipoyllysine-residue acetyltransferase component 1 of pyruvate dehydrogenase complex | W9SF29 | 4 | 67928.33 | 5.33 |  |
| Cytochrome c oxidase subunit 5b-2 | W9QF69 | 3 | 18284.22 | 5.11 |  |
| Isocitrate dehydrogenase [NAD] subunit, mitochondrial | A0A0K0PUB0 | 3 | 41387.76 | 6.64 |  |
| Gamma-aminobutyrate transaminase | J9XGP8 | 3 | 56662.15 | 6.74 |  |
| Citrate synthase | W9QEE8 | 3 | 52995.52 | 6.87 |  |
| Monodehydroascorbate reductase | W9QH91 | 3 | 53244.86 | 8.11 |  |
| Cysteine desulfurase 1 | W9RYP8 | 3 | 50448.19 | 6.85 |  |
| Cytochrome c1-1, heme protein | W9SAX0 | 3 | 68680.16 | 8 |  |
| V-type proton ATPase catalytic subunit A | W9S238 | 3 | 68751.7 | 5.33 |  |
| Gamma-aminobutyrate transaminase 2 | J9XGZ5 | 3 | 56732.19 | 7.24 |  |
| Histone H2B | M5W0R4 | 3 | 16023.58 | 10.05 |  |
| Mitochondrial Rho GTPase | M5W3L4 | 3 | 71815.88 | 5.41 |  |
| Acyl carrier protein | M5WHZ2 | 3 | 14239.12 | 5.21 |  |
| Sulfurtransferase | M5X0Q0 | 3 | 34847.82 | 5.51 |  |
| Glutamate dehydrogenase | M5X1C9 | 3 | 44731.51 | 6.33 |  |
| ATPase family AAA domain-containing protein 3 | W9R6N2 | 3 | 70271.67 | 9.37 |  |
| Glycine dehydrogenase decarboxylating protein | W8SQT8 | 3 | 114024.1 | 6.6 |  |
| Acetyltransferase component of pyruvate dehydrogenase complex | W9QG40 | 3 | 55952 | 6.74 |  |
| Elongation factor Tu | W9QVI5 | 3 | 51596.95 | 7.67 |  |
| Chaperone protein | W9QX74 | 3 | 110006.3 | 6.55 |  |
| Mitochondrial outer membrane protein porin of 34 kDa | W9QZM5 | 3 | 29615.99 | 8.56 |  |
| CBS domain-containing protein CBSX3 | W9R5S5 | 3 | 52652.99 | 9.5 |  |
| Succinate-semialdehyde dehydrogenase (Acetylating) | W9RCD5 | 3 | 49261.93 | 8.38 |  |
| NADH dehydrogenase [ubiquinone] flavoprotein 1 | W9RVY5 | 3 | 45015.17 | 7.11 |  |
| Methylglutaconyl-CoA hydratase | W9RXQ9 | 3 | 70522.33 | 5.01 |  |
| Aspartate aminotransferase | W9RZ69 | 3 | 42706.5 | 8.32 |  |
| ATP synthase subunit gamma | M5WB68 | 2 | 35215.27 | 9.12 |  |
| ATP synthase subunit delta | W9QU14 | 2 | 21507.31 | 6.07 |  |
| Mitochondrial outer membrane protein porin of 36 kDa | W9R9M3 | 2 | 29435.75 | 7.86 |  |
| Hexaprenyldihydroxybenzoate methyltransferase | W9QLG7 | 2 | 35604.13 | 6.19 |  |
| NADH dehydrogenase [ubiquinone] flavoprotein 2 | W9R3Y3 | 2 | 35692.59 | 9.25 |  |
| Cytochrome b-c1 complex subunit Rieske, mitochondrial | W9RBL2 | 2 | 30406.31 | 8.98 |  |
| Putative aminotransferase y4uB | W9RF04 | 2 | 51418.24 | 5.55 |  |
| Stroma ascorbate peroxidase | A0A067XJ37 | 2 | 42048.91 | 7.76 |  |
| L-galactono-1,4-lactone dehydrogenase (Fragment) | A0A0H4LTI6 | 2 | 45503.61 | 6.48 |  |
| Aquaporin | D8V5R9 | 2 | 30261.82 | 8.73 |  |
| NADH dehydrogenase subunit 2 | J7MFW9 | 2 | 53507.56 | 8.97 |  |
| Poly [ADP-ribose] polymerase | M5VIF4 | 2 | 71219.19 | 6.89 |  |
| Peptidyl-prolylcis-trans isomerase | M5W0F8 | 2 | 18855.32 | 8.31 |  |
| Fructose-bisphosphate aldolase | M5W2H9 | 2 | 38412.33 | 6.92 |  |
| 40S ribosomal protein | W9S6T6 | 2 | 23004.31 | 9.85 |  |
| Iron-sulfur cluster assembly protein | M5WAY2 | 2 | 18519.99 | 8.65 |  |
| Mitochondrial phosphatic carrier | M5WU44 | 2 | 39665.8 | 9.35 |  |
| Dihydroorotate dehydrogenase (quinone), mitochondrial | M5WGE5 | 2 | 48794.3 | 9.17 |  |
| Lon protease homolog | M5WHA3 | 2 | 109877.8 | 5.31 |  |
| 3-hydroxyisobutyrate dehydrogenase | M5WHG3 | 2 | 37271.01 | 6.6 |  |
| Ras-related protein | W9R1W6 | 2 | 23149.97 | 6.96 |  |
| Malic enzyme | M5X6Q4 | 2 | 70223.54 | 5.95 |  |
| Eukaryotic translation initiation factor 5A | Q2L998 | 2 | 17132.07 | 5.3 |  |
| Glutathione peroxidase | Q6A4W8 | 2 | 18530.81 | 6.13 |  |
| Catalase | Q7XTK8 | 2 | 57038.6 | 6.95 |  |
| Preprofigain B (Fragment) | S4TA71 | 2 | 41459.94 | 5.7 |  |
| 10 kDachaperonin | W9QG08 | 2 | 10617.24 | 8.92 |  |
| NifU-like protein 4 | W9QGE4 | 2 | 31759.33 | 5.1 |  |
| Protein usf | W9QIC5 | 2 | 29250.21 | 9.14 |  |
| Glutathione gamma-glutamylcysteinyltransferase 3 | W9QSW3 | 2 | 55287.96 | 5.65 |  |
| Peroxiredoxin-2F | W9QVC2 | 2 | 22479.44 | 8.91 |  |
| Putative NADH-ubiquinone oxidoreductase | W9QEJ3 | 2 | 74300.17 | 9.02 |  |
| Hexokinase | W9R6M8 | 2 | 54076.36 | 5.64 |  |
| Histone | W9RHQ2 | 2 | 22064.46 | 10.91 |  |
| Methylcrotonoyl-CoA carboxylase subunit alpha | W9RHZ1 | 2 | 127791.6 | 7.45 |  |
| ATP-dependent Clp protease ATP-binding subunit ClpX | W9RJ61 | 2 | 41905.67 | 8.56 |  |
| 3-ketoacyl-CoA thiolase 2 | W9RJR4 | 2 | 48666.32 | 8.59 |  |
| Aminomethyltransferase | W9RJW9 | 2 | 44059.97 | 8.9 |  |
| Adenylate kinase | W9RSI3 | 2 | 26430.26 | 7.65 |  |
| 3-hydroxyisobutyrate dehydrogenase | W9RWB3 | 2 | 36831.14 | 8.94 |  |
| ABC transporter G family member | W9S228 | 2 | 148234 | 8.59 |  |
| Methylcrotonoyl-CoA carboxylase beta chain | W9S685 | 2 | 45677.62 | 6.38 |  |
| Succinate dehydrogenase [ubiquinone] iron-sulfur subunit, mitochondrial | W9S8I7 | 2 | 31393.95 | 8.86 |  |
| Isocitrate dehydrogenase [NAD] regulatory subunit 1 | W9S8T8 | 2 | 39908.6 | 8.41 |  |
| Long chain acyl-CoA synthetase 7 | W9SMR0 | 2 | 75881.15 | 7.52 |  |
| Mitochondrial Rho GTPase | W9T1N4 | 2 | 72016.13 | 5.68 |  |
| Superoxide dismutase [Mn], mitochondrial | Q9SM64 | 2 | 25454.71 | 8.62 |  |
| Triosephosphate isomerase | M5VZS0 | 1 | 27321.95 | 5.56 |  |
| Cysteine synthase | M5X090 | 1 | 40689.6 | 8.78 |  |
| Glycine-rich RNA-binding protein 2 | W9QTX1 | 1 | 15545.02 | 7.84 |  |
| 60S acidic ribosomal protein | Q8H2B9 | 1 | 11414.63 | 4.3 |  |
| Mitochondrial import inner membrane translocase subunit TIM44 | W9RRU8 | 1 | 54152.56 | 8.71 |  |
| GrpE protein homolog | M5WTU3 | 1 | 37117.95 | 6.29 |  |
| globulin isoform 2 | T1WP68 | 1 | 79764.85 | 6.35 |  |
| ACC oxidase | L0ARH8 | 1 | 36807.62 | 4.95 |  |
| Kinesin-like protein | M5VLU3 | 1 | 333296.8 | 5.16 |  |

**Table S3 Zn^2+^ -IMAC captured metal-binding proteins detected by LC-MS/MS.**

| **Description** | **Accession number** | **UniquePepCount** | **MW** | **pI** |
| --- | --- | --- | --- | --- |
| ATP synthase F1 subunit 1 | A0A0M5M1Z3 | 21 | 55322.9 | 5.84 |
| ATP synthase subunit alpha | A0A0F6T7P4 | 19 | 53667.81 | 6.22 |
| Chaperonin CPN60-2 | W9SB24 | 19 | 61276.96 | 5.65 |
| ADP,ATP carrier protein 3 | W9QNM9 | 14 | 40398.07 | 9.7 |
| ATP synthase subunit beta | W9R2H6 | 13 | 59321.85 | 5.83 |
| Heat shock protein 60 (Fragment) | Q8H6U4 | 13 | 57762.96 | 5.26 |
| Lon protease homolog | W9SNF1 | 13 | 107972.7 | 5.63 |
| Heat shock 70 kDa protein | W9SKK5 | 13 | 71725.46 | 5.94 |
| ATP-dependent zinc metalloprotease FTSH 10 | W9RUG9 | 13 | 90641.94 | 6.98 |
| NADH dehydrogenase subunit 7 | A0A0B4L024 | 12 | 44602.48 | 6.71 |
| Dihydrolipoyl dehydrogenase | W9R882 | 12 | 52744.14 | 6.9 |
| Lon protease homolog | M5WHA3 | 12 | 109877.8 | 5.31 |
| V-type proton ATPase catalytic subunit A | W9S238 | 12 | 68751.7 | 5.33 |
| ATPase family AAA domain-containing protein 3 | W9R6N2 | 11 | 70271.67 | 9.37 |
| Heat shock protein 90 | W9SBQ7 | 11 | 90068.66 | 5.16 |
| Elongation factor Tu | W9QVI5 | 10 | 51596.95 | 7.67 |
| Malic enzyme | W9QXC3 | 10 | 71645.76 | 6.42 |
| Mitochondrial Rho GTPase | W9T1N4 | 10 | 72016.13 | 5.68 |
| NADH dehydrogenase [ubiquinone] iron-sulfur protein 1 | W9RU22 | 9 | 81677.63 | 6.64 |
| Serine hydroxymethyltransferase | M5XYL0 | 9 | 57134.58 | 8.11 |
| V-ATPase subunit B1 | A0A0B4S3Q7 | 9 | 54586.44 | 5.03 |
| Catalase | G9JJS6 | 9 | 57011.57 | 6.78 |
| Aldehyde dehydrogenase family 2 member | W9RMT5 | 9 | 59392.13 | 8.3 |
| Mitochondrial outer membrane protein porin of 36 kDa | W9R9M3 | 8 | 29435.75 | 7.86 |
| Succinate dehydrogenase [ubiquinone] flavoprotein subunit, mitochondrial | W9SD93 | 8 | 68781.74 | 6.46 |
| 2-oxoglutarate dehydrogenase | W9R2F3 | 8 | 115922.2 | 6.62 |
| Mitochondrial uncoupling protein 3 | W9S5K1 | 7 | 39064.66 | 9.49 |
| Aconitatehydratase | M5XA02 | 7 | 108704.3 | 6.98 |
| NADH dehydrogenase subunit 9 | A0A0M4R4N3 | 6 | 22667.22 | 6.53 |
| Formate dehydrogenase | W9SX88 | 6 | 42196.64 | 6.68 |
| Elongation factor 1-alpha | M5W5P0 | 6 | 49472.72 | 9.15 |
| Chaperone protein | W9QX74 | 6 | 110006.3 | 6.55 |
| L-galactono-1,4-lactone dehydrogenase | W9RS52 | 6 | 68145.93 | 8.77 |
| Voltage-dependent anion channel protein | A0A068F674 | 5 | 29806.16 | 7.88 |
| Dihydrolipoyllysine-residue succinyltransferase component of 2-oxoglutarate dehydrogenase complex 2 | W9R4B7 | 5 | 46470.35 | 8.66 |
| Isocitrate dehydrogenase [NAD] subunit, mitochondrial | M5VYP8 | 5 | 41338.72 | 6.61 |
| Mitochondrial-processing peptidase subunit alpha | W9RH22 | 5 | 54235 | 6.1 |
| Putative mitochondrial-processing peptidase subunit beta | W9QID6 | 5 | 59112.28 | 6.17 |
| Malate dehydrogenase | W9S9W9 | 5 | 36489.57 | 9 |
| NADH-ubiquinone oxidoreductase chain 5 | A0A0F6QRP7 | 5 | 66865.29 | 7.97 |
| Methylcrotonoyl-CoA carboxylase beta chain | W9S685 | 5 | 45677.62 | 6.38 |
| ATP synthase subunit d | W9SNX9 | 5 | 19717.04 | 5.1 |
| Pyruvate dehydrogenase E1 component subunit alpha | W9RNK6 | 5 | 35897.7 | 7.11 |
| Arginase | B3F0K4 | 5 | 37000.83 | 6.14 |
| Succinyl-CoA ligase subunit beta | M5VJR8 | 5 | 45271.83 | 5.86 |
| Acetyltransferase component of pyruvate dehydrogenase complex | W9QG40 | 5 | 55952 | 6.74 |
| Adenylate kinase | W9RSI3 | 5 | 26430.26 | 7.65 |
| Leucineaminopeptidase | W9RX96 | 5 | 60314.29 | 6.02 |
| Aminomethyltransferase | W9RJW9 | 4 | 44059.97 | 8.9 |
| Prohibitin-1 | W9S8G3 | 4 | 30445.49 | 8.86 |
| Mitochondrial phosphatic carrier | M5WU44 | 4 | 39665.8 | 9.35 |
| Iron-sulfur cluster assembly protein | M5WAY2 | 4 | 18519.99 | 8.65 |
| Serine hydroxymethyltransferase | W9RZN3 | 4 | 39139.46 | 8.68 |
| Dihydroorotate dehydrogenase (quinone), mitochondrial | M5WGE5 | 4 | 48794.3 | 9.17 |
| Calcium-transporting ATPase | M5XQ66 | 4 | 114074.9 | 6.97 |
| Putative aquaporin PIP1-4 | W9QHC5 | 4 | 30704.28 | 8.9 |
| V-type proton ATPase subunit a | A0A0B4S3H9 | 4 | 95084.31 | 5.79 |
| Nucleoside diphosphate kinase | M5WAF8 | 4 | 25804.14 | 9.18 |
| Glutamate dehydrogenase | M5X1C9 | 4 | 44731.51 | 6.33 |
| Isovaleryl-CoA dehydrogenase 1 | W9RMP4 | 4 | 41471.14 | 7.46 |
| Calnexin-1-like protein | W9S9M2 | 4 | 62487.21 | 4.66 |
| DNA gyrase subunit A | W9SBT9 | 4 | 112571.3 | 9 |
| Succinate dehydrogenase [ubiquinone] iron-sulfur subunit, mitochondrial | M5WSZ8 | 4 | 39363.32 | 9.1 |
| Hydroxymethylglutaryl-CoA lyase | W9SJS1 | 4 | 37644.94 | 8.94 |
| Mitochondrial outer membrane protein porin 4 | W9T279 | 3 | 37705.49 | 9.18 |
| Aspartic proteinase | W9RRG4 | 3 | 56072.08 | 5.12 |
| Aldehyde dehydrogenase family 6 member B2 | W9SA81 | 3 | 104155.1 | 8.48 |
| ATP synthase subunit gamma | M5WB68 | 3 | 35215.27 | 9.12 |
| Isocitrate dehydrogenase [NAD] regulatory subunit 1 | W9S8T8 | 3 | 39908.6 | 8.41 |
| Hexokinase | W9SH10 | 3 | 41880.83 | 5.9 |
| Electron transfer flavoprotein-ubiquinone oxidoreductase | W9QWG9 | 3 | 109357.4 | 8.37 |
| Succinate-semialdehyde dehydrogenase | W9RCD5 | 3 | 49261.93 | 8.38 |
| Putative aminotransferase y4uB | W9RF04 | 3 | 51418.24 | 5.55 |
| Mechanosensitive ion channel protein 1 | W9SK27 | 3 | 59463.21 | 9.27 |
| V-ATPase subunit E1 | A0A0A7G0Q3 | 3 | 26088.74 | 7.06 |
| Cytochrome c oxidase subunit 3 | A0A0B4L074 | 3 | 29555.07 | 6.92 |
| NADH dehydrogenase subunit 2 | A0A0B4L0G8 | 3 | 54789.06 | 8.97 |
| Ribosomal protein L16 | A0A0M4S8G8 | 3 | 16077.52 | 11.16 |
| Sulfurtransferase | M5X0Q0 | 3 | 34847.82 | 5.51 |
| ATP-dependent Clp protease proteolytic subunit | M5XT83 | 3 | 26704.33 | 7.78 |
| Long chain acyl-CoA synthetase 8 | W9QFE0 | 3 | 83274.96 | 8.8 |
| Putative NADH dehydrogenase [ubiquinone] 1 alpha subcomplex subunit 12 | W9QKF5 | 3 | 41541.06 | 9.51 |
| Solanesyldiphosphate synthase 3 | W9QTA8 | 3 | 75552.06 | 6.42 |
| NADH-cytochrome b5 reductase | W9QYB4 | 3 | 30727.11 | 7.09 |
| Heat shock 70 kDa protein 4 | W9R5Z5 | 3 | 71197.77 | 5.07 |
| [Pyruvate dehydrogenase [lipoamide]] kinase | W9RL69 | 3 | 43952.24 | 8.77 |
| Cysteine desulfurase 1 | W9RYP8 | 3 | 50448.19 | 6.85 |
| 40S ribosomal protein S3-3 | W9RRF5 | 3 | 31735.94 | 9.46 |
| Putative NADH dehydrogenase | W9RUS8 | 3 | 55300.87 | 7.23 |
| Sorting and assembly machinery component 50-B-like protein | W9RRI7 | 3 | 60727.02 | 5.55 |
| Aspartate aminotransferase | W9RZ69 | 3 | 42706.5 | 8.32 |
| Formyltetrahydrofolatedeformylase | W9SED1 | 3 | 44708.1 | 9.3 |
| Dihydrolipoyllysine-residue acetyltransferase component 1 of pyruvate dehydrogenase complex | W9SF29 | 3 | 67928.33 | 5.33 |
| 50S ribosomal protein | W9SMR4 | 3 | 18487.26 | 9.02 |
| Acetyltransferase component of pyruvate dehydrogenase complex | M5XCC8 | 2 | 58798 | 8.03 |
| ATPase subunit 8 | A0A0M4R6S6 | 2 | 18230.83 | 9.08 |
| Mitochondrial import inner membrane translocase subunit TIM44 | W9RRU8 | 2 | 54152.56 | 8.71 |
| DUF21 domain-containing protein | W9RVI7 | 2 | 44448.95 | 5.76 |
| NADH-ubiquinone oxidoreductase chain 1 | A0A0B4L2C5 | 2 | 32563.39 | 9.44 |
| 3-oxoacyl-[acyl-carrier-protein] synthase | M5WUQ7 | 2 | 49138.27 | 6.6 |
| DNA gyrase subunit B | W9QCV3 | 2 | 83057.05 | 8.04 |
| ATP synthase subunit delta | W9QU14 | 2 | 21507.31 | 6.07 |
| Peptide transporter | W9SG60 | 2 | 124386.8 | 5.28 |
| Ribosomal protein S7 | A0A0B4KZZ7 | 2 | 17099.82 | 10.45 |
| NADH-ubiquinone oxidoreductase chain 6 | A0A0B4L268 | 2 | 22597.9 | 9.9 |
| NADH-ubiquinone oxidoreductase chain 5 | E5DLC2 | 2 | 40329.76 | 8.48 |
| NADPH:adrenodoxin oxidoreductase, mitochondrial | M5W1W1 | 2 | 53443.35 | 7.19 |
| Uridine kinase | M5XJT9 | 2 | 74230.59 | 6.56 |
| 60S ribosomal protein L12 | W9R6G5 | 2 | 17882.58 | 9.02 |
| Glycine dehydrogenase decarboxylating protein | W8SQT8 | 2 | 114024.1 | 6.6 |
| Delta-1-pyrroline-5-carboxylate dehydrogenase 12A1 | W9QIA3 | 2 | 71852.25 | 7.65 |
| Monosaccharide-sensing protein 2 | W9QTX1 | 2 | 79329.23 | 5.28 |
| Putative copper-transporting ATPase PAA1 | W9QVF9 | 2 | 100340.9 | 8.79 |
| External NADH-ubiquinone oxidoreductase 1 | W9R003 | 2 | 63840.27 | 8.94 |
| NADH dehydrogenase [ubiquinone] flavoprotein 2 | W9R3Y3 | 2 | 35692.59 | 9.25 |
| Protein disulfide-isomerase | W9R743 | 2 | 56240.96 | 4.92 |
| NADH dehydrogenase [ubiquinone] iron-sulfur protein 4 | W9RS61 | 2 | 17355.37 | 9.87 |
| Glyceraldehyde-3-phosphate dehydrogenase | W9RTC6 | 2 | 36916.73 | 7.7 |
| Fumaratehydratase 1 | W9RTY2 | 2 | 53269.23 | 6.84 |
| Methylglutaconyl-CoA hydratase | W9RXQ9 | 2 | 70522.33 | 5.01 |
| Pentatricopeptide repeat-containing protein | W9QZM9 | 2 | 89228.44 | 8.98 |
| 2-oxoglutarate/malate translocator | W9S711 | 2 | 59676.69 | 9.72 |
| Citrate synthase | I3VKW1 | 1 | 52575.9 | 7.25 |
| Biotin synthase | W9RFB7 | 1 | 41481 | 7.57 |
| Alanine aminotransferase | W9RA79 | 1 | 59032.42 | 5.91 |
| Ornithine aminotransferase | W9QV49 | 1 | 32311.12 | 5.71 |
| 11S globulin isoform 3A | A1E0V7 | 1 | 56989.33 | 8.8 |
| Kinesin-like protein | M5WKW6 | 1 | 76836.27 | 8.97 |
| V-type proton ATPase subunit | W9QL95 | 11 | 62916.4 | 5.13 |

**Table S4 Fe^2+^ -IMAC captured metal-binding proteins detected by LC-MS/MS.**

| **Description** | **Accession number** | **UniquePepCount** | **MW** | **pI** |
| --- | --- | --- | --- | --- |
| ATP synthase F1 subunit 1 | A0A0M5M1Z3 | 26 | 55322.9 | 5.84 |
| Uncharacterized protein | M5XYE7 | 23 | 61197.76 | 5.62 |
| ATP synthase subunit alpha | A0A0B4L0T2 | 23 | 54966.25 | 5.84 |
| Chaperonin CPN60-2 | W9SB24 | 20 | 61276.96 | 5.65 |
| Uncharacterized protein | M5X833 | 18 | 115797.7 | 6.51 |
| 2-oxoglutarate dehydrogenase | W9R2F3 | 18 | 115922.2 | 6.62 |
| Heat shock protein 60 | Q8H6U4 | 16 | 57762.96 | 5.26 |
| ATP synthase subunit beta | W9R2H6 | 15 | 59321.85 | 5.83 |
| Chaperone protein | W9QX74 | 14 | 110006.3 | 6.55 |
| ADP,ATP carrier protein 3 | W9QNM9 | 13 | 40398.07 | 9.7 |
| Heat shock 70 kDa protein | W9SKK5 | 13 | 71725.46 | 5.94 |
| Lon protease homolog | W9SNF1 | 12 | 107972.7 | 5.63 |
| Aldehyde dehydrogenase family 2 member | W9RMT5 | 11 | 59392.13 | 8.3 |
| NADH dehydrogenase [ubiquinone] iron-sulfur protein 1 | W9RU22 | 11 | 81677.63 | 6.64 |
| Aconitatehydratase | W9RLC6 | 11 | 102299.8 | 8.07 |
| Succinyl-CoA ligase subunit beta | W9R0G6 | 10 | 45263.87 | 6.07 |
| Heat shock protein 90 | W9SBQ7 | 10 | 90068.66 | 5.16 |
| NADH dehydrogenase subunit 7 | A0A0B4 | 9 | 44602.48 | 6.71 |
| V-ATPase subunit B1 | A0A0B4S3Q7 | 9 | 54586.44 | 5.03 |
| ATP synthase subunit d | W9SNX9 | 8 | 19717.04 | 5.1 |
| Mitochondrial outer membrane protein porin of 36 kDa | W9R9M3 | 7 | 29435.75 | 7.86 |
| Succinate dehydrogenase [ubiquinone] flavoprotein subunit | W9SD93 | 7 | 68781.74 | 6.46 |
| Dihydrolipoyl dehydrogenase | W9R882 | 6 | 52744.14 | 6.9 |
| ATPase family AAA domain-containing protein 3 | W9R6N2 | 6 | 70271.67 | 9.37 |
| Cysteine desulfurase 1 | W9RYP8 | 5 | 50448.19 | 6.85 |
| Mitochondrial outer membrane protein porin of 34 kDa | W9QZM5 | 5 | 29615.99 | 8.56 |
| Voltage-dependent anion channel protein | A0A068F674 | 4 | 29806.16 | 7.88 |
| ATP-dependent Clp protease proteolytic subunit | M5XT83 | 4 | 26704.33 | 7.78 |
| Mitochondrial uncoupling protein 3 | W9S5K1 | 3 | 39064.66 | 9.49 |
| Sulfurtransferase | M5X0Q0 | 3 | 34847.82 | 5.51 |
| Mitochondrial import receptor subunit TOM40-1-like protein | W9RWE4 | 3 | 34243.74 | 7.09 |
| Protein disulfide-isomerase | W9R743 | 3 | 56240.96 | 4.92 |
| Cytochrome c1-1, heme protein | W9SAX0 | 3 | 68680.16 | 8 |
| NADPH:adrenodoxin oxidoreductase, mitochondrial | M5W1W1 | 3 | 53443.35 | 7.19 |
| Cytochrome c oxidase subunit 5b-2 | W9QF69 | 3 | 18284.22 | 5.11 |
| NADH-cytochrome b5 reductase | M5W2F6 | 2 | 35652.78 | 8.67 |
| Iron-sulfur cluster assembly protein | M5WAY2 | 2 | 18519.99 | 8.65 |
| Citrate synthase | W9QEE8 | 2 | 52995.52 | 6.87 |
| Glutathione peroxidase | W9QH65 | 2 | 26518.93 | 9.34 |
| Heat shock protein 83 | W9QH95 | 2 | 80735.77 | 5.01 |
| Cytochrome c1-1, heme | W9QQ48 | 2 | 32448.38 | 5.73 |
| ATP-dependent Clp protease ATP-binding subunit ClpX | W9RLE1 | 2 | 31405.54 | 9.32 |
| Mitochondrial import inner membrane translocase subunit TIM50 | W9QMZ2 | 2 | 40905.25 | 9 |
| DUF21 domain-containing protein | W9RVI7 | 1 | 44448.95 | 5.76 |
| Kinesin-like protein | M5W7Z3 | 1 | 125248.6 | 8.7 |
